# Efficacy of functional endoscopic sinus surgery for symptoms in chronic rhinosinusitis with or without polyposis

**DOI:** 10.1016/S1808-8694(15)30062-8

**Published:** 2015-10-19

**Authors:** Débora Lopes Bunzen, Alexandre Campos, Fernando Souza Leão, Alberto Morais, Fabiana Sperandio, Silvio Caldas Neto

**Affiliations:** a Third year resident.; b Third year resident.; c Otorhinolaryngologist.; d Otorhinolaryngologist.; e PhD in Otorhinolaryngology, preceptor at the Otorhinolaryngology Department - University Hospital - UFPE.; f Associate Professor of Otorhinolaryngology - University Hospital - UFPE.

**Keywords:** Sinusitis, Symptoms, Funcional endoscopi sinus surgery, Quality of life

## Abstract

Functional endoscopic sinus surgery is the preferred treatment for chronic rhinosinusitis currently. Success on symptoms relief and quality of life improvement are the study leading objectives. **Study design:** retrospective clinical trial. **Methods:** Questionnaires were given to the patients referred to Hospital das Clinicas-UFPE to chronic rhinosinusitis (CRS) functional endoscopic sinus intervention during 2003-2004. Symptoms outcome before and after surgery were compared and analyzed using a five-point-ranking scale. **Results:** Twenty-four pacients answered the questions. Eleven pacientes had CRS and 13 had CRS associated with nasal polypos. Quality of life was restricted by CRS in everyone, with a good improvement in 54,2% cases. All patients could recommend functional endoscopic sinus surgery to someone with same nasal problems and only 3 would not get surgery again. The best symptoms relif results were: nasal obstruction (83,3%), cacosmia/halitosis (80%), hyposmia/anosmia (63,15%), headache (62%). Patients with polyps achieved better symptomatic response than patients with only CRS. **Conclusions:** The leading simptoms were improved by functional endoscopic sinus surgery but not so much we expect. Allergic rhinits presenting, not using nasal spray, poor ambient control influenced this result. Polyps patient achivied better symptoms outcome and quality of life responses on the most of symptoms than CRS pacients.

## INTRODUCTION

Currently, endoscopic sinus surgery is seen as the standard treatment in clinically challenging chronic rhinosinusitis (CRS) and in nasosinusal polyposis. The endoscopic procedure is based on the principles introduced by Messerklinger1, which prioritize both the function and the permeability of pre-ethmoidal spaces, in a precise and guided intervention on the lateral wall of the nose, thus bringing about good ventilation and drainage for facial sinuses. In 1985, Kennedy^2^ described this technique and popularized the term “Functional Endoscopic Sinus Surgery - FESS”. Since then, there have been a number of progresses in both the technique and surgery indication.

There are a number of papers in the literature about the efficacy of the endoscopic surgery, reports of its capabilities and questions about its true worth^3^, ^4^. Notwithstanding, in order to assess how efficient a treatment method is, we have, first of all, to define the pathology for which the treatment will be used. In our study we assessed FESS efficiency in mitigating, or even abolishing symptoms related to CRS alone or associated with nasosinusal polyposis, through a questionnaire bearing simple questions and direct answers on the severity of symptoms presented by such patients.

## METHODOLOGY

Our study encompassed 24 patients who answered the questionnaire proposed and underwent FEES in the years of 2003 and 2004. Of this total, 11 were diagnosed with CRS and 13 with CRS associated with nasosinusal polyposis. We excluded recurrences and reoperation cases. The diagnosis of both pathologies was clinical and radiological; and the clinical treatment attempted for CRS was based on large spectrum antibiotics against upper airway infections and topical steroids, as nasal sprays, for at least twelve weeks.

All the endoscopic procedures were carried out in the University Hospital of the Federal University of Pernambuco, under general anesthesia, by second and third year residents under supervision. According to the protocol in our service, the surgical technique used was the one described by Messerklinger1 and Stammberger^5^. Average hospital stay was 24 hours, with post-operative follow up in 7, 15, 30 days, and in 6 months. Cephalexin was used for 7 days in the post-op, and the patients were instructed to vigorously spray 0.9% saline solution in their noses. Topical steroid was started 15 days after the procedure, and continued if necessary. Patients with nasosinusal polyps also used Betamethasone for 10 days prior to surgery and 20mg/day of prednisolone for 7 days after the procedure. Nasal cleaning and crust removal were carried out in the post op follow up.

All these patients answered questionnaires about their symptoms and their life quality before and after surgery (Attachment 1). 12 questions were prepared about pre-op symptoms and 15 about post-op symptoms. We were careful enough to use simple language in preparing the questions so that all the patients would understand them. Each answer was scored from 0 to 5 according to Likert’s scale^6^, as depicted in the attached questionnaire. The questionnaire was adapted from the literature7 and the information generated compared CRS alone and CRS with polyps patients.

Attachment 1 **Questionnaire**

Name:

Data of surgery:

Indication:   ( ) polyps  ( ) chronic sinusitis

1 - How does the disease affect your life quality?
  ( ) Does not affect ( ) a little ( ) somewhat ( ) much ( ) incapacitates
2 - How much does the disease obstruct your nose?
  ( ) Does not obstruct ( ) a little ( ) somewhat ( ) much ( ) incapacitates
3 - How about headaches?
  ( ) Does not have ( ) a little ( ) somewhat ( ) much ( ) incapacitates
4 - How about olfaction?
  ( ) Does not affect ( ) a little ( ) somewhat ( ) much ( ) incapacitates
5 - How about secretion?
  ( ) Does not affect ( ) a little ( ) somewhat ( ) much ( ) incapacitates
6 - How about dry nose or dry mouth?
  ( ) Does not have ( ) a little ( ) somewhat ( ) much ( ) incapacitates
7 - How about cough?
  ( ) Does not have ( ) a little ( ) somewhat ( ) much ( ) incapacitates
8 - How about asthma?
  ( ) Does not have ( ) a little ( ) somewhat ( ) much ( ) incapacitates
9 - How about post nasal discharge?
  ( ) Does not have ( ) a little ( ) somewhat ( ) much ( ) incapacitates
10- How about foul smell or bad breath?
  ( ) Does not have ( ) a little ( ) somewhat ( ) much ( ) incapacitates
  The following questions are only for patients who have already been operated:
11- If your disease returned, would you operate again?
  ( ) Yes   ( ) no
12- Would you recommend this procedure to another person?
  ( ) Yes   ( ) no

In order to analyze the data, we considered good surgery outcome when the score difference between pre and post operative symptoms for each variable resulted in a positive value equal or above 2. If the difference resulted in a negative value, equal or above 2, the procedure was considered a failure. In cases which the score difference between pre and post op were between +1 or -1, we concluded that the procedure did not influence a certain symptom.

All patients signed an informed consent approved by the Hospital Ethics Committee and were duly explained about the study goals, and their personal information was not disclosed.

## RESULTS

Of all the patients who answered the questionnaire, 10 were male and 14 were female, all young adults. They were then separated in two groups, the ones of CRS alone were 11 in total and those with CRS and polyps were 13. As for health improvement after the procedure, in both groups 50% reported excellent outcome. Life quality was impaired in all interviewed patients, and it improved considerably in 54.2% of the cases. All those interviewed patients would recommend the procedure to other people with similar nasosinusal problems and only 3 patients would not undergo the surgery again if the problem recurred.

Table 1 lists the symptoms reported by patients with CRS and CRS with nasosinusal polyps. Table 2 depicts the results obtained through FESS surgical success criteria described in the methodology.

**Table 1:** Symptoms prevalence found in patients who underwent functional endoscopic sinus surgery to treat chronic rhinosinusitis and nasal polyps.

Symptom	CRS	CRS and Polyps
Nasal obstruction	11 (100%)*	13 (100%)
Headaches	11 (100%)	10(77%)
Hyposmia/anosmia	7 (63,5%)	12 (92,3%)
Secretion	9 (82%)	13 (100%)
Dry throat	5 (45,5%)	13 (100%)
Cough	4 (36,3%)	9 (69,2%)
Asthma	3 (27,2%)	5 (38,5%)
Post-nasal drip	8 (72,7%)	11 (84,6%)
Cacosmia and halitosis	6 (54,5%)	4 (30,7%)

* The percentage was calculated based on the total number of patients with CRS in the first column and the total number of patients with CRS and polyps in the second column.

**Table 2:** General success rates achieved in treating symptoms with FESS reported by the patients.

	Improved*	No change*	Got worse	Without the symptom
Nasal obstruction	20 (83,3%)	4 (16,7%)	0	0
Headaches	13(61,9%)	8 (38,1%)	0	3
Hyposmia/anosmia	12 (63,9%)	7 (36,1%)	1	4
Secretion	10(45,4%)	12 (55,6%)	0	2
Dry throat	10(55,5%)	8 (44,5%)	0	6
Cough	4 (30,7%)	9 (69,3%)	1	10
Asthma	2 (25,0%)	6 (75,0%)	0	16
Post-nasal drip	9 (47,3%)	10 (52,7%)	0	5
Cacosmia and halitosis	8 (80%)	2 (20%)	0	14

* The percentage was calculated based on the total number of patients who presented each specific symptom.

The most prevalent symptoms were: nasal obstruction, headaches (present in all patients with CRS), secretion and dry throat (present in all patients with CRS and polyps). We compared symptoms improvement in both CRS and CRS with nasal polyps. Hyposmia was present in 7 patients with CRS, with improvement in 4 cases (57.1%). In CRS and polyps we had improvement in 8 of the 12 patients (66.6%).

**Graph 1:**
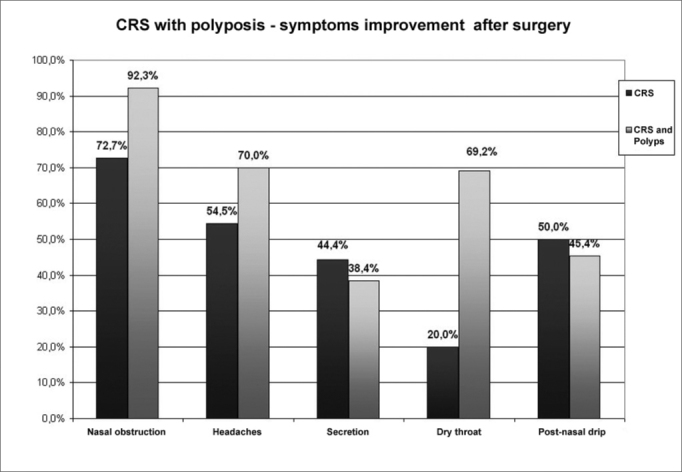

Graph 1 compares most prevalent symptoms improvement in CRS and CRS with polyps.

## DISCUSSION

Chronic rhinosinusitis is one of the most common diseases in the United States. It is estimated that 15% of the American population has CRS^8^. This pathology is based on the chronic inflammation of the paranasal sinuses mucosa, with secretion stasis and bacterial infection. The Brazilian consensus has defined it as an inflammatory process in the mucosa that lines the nasal cavity and paranasal sinuses, with symptoms remaining for over 12 weeks. CRS symptoms may range from minor to major. The major symptoms are nasal obstruction, a feeling of facial pressure, rhinorrhea and hyposmia; and the minor symptoms are: headache, toothache, halitosis, fatigue, dry cough, fever and earache^9^, whereas 2 major symptoms, one major and 2 minors or purulent discharge in the nasal cavity suggest CRS.

Nasosinusal polyps are the most frequent indication for nasosinusal endoscopic surgical intervention, after CRS. The cause of polyp formation is still uncertain. There are theories based on edematous alterations in the nasal mucosa, and others that advocate the participation of fungi in the physiopathology of this disease. The nasal polyp may be single, clearly outlined or involving all the facial sinuses, being thus characterized as nasosinusal polyposis or diffuse polyposis. The staging proposed by Stamm is based on four levels of disease^10^. The most frequent sites for polyps are the anterior spaces of the ethmoid sinus where the symptoms reported by patients are very much similar to those reported by CRS patients - nasal obstruction is the major complaint^11^.

Both CRS and nasal polyps diagnosis are based on clinical criteria. The purely subjective symptoms, described by the patients, should guide the medical treatment, as far as for complementary tests to be ordered - CT scan is the foremost, therapeutic to be used. Thus, any study dedicated to measure the efficacy of nasal endoscopic surgery for these pathologies should be based on symptoms. The patients’ complaints and how much the disease is limiting their daily activities should be the focus of such analysis. In our department, those patients who fulfill the clinical criteria for CRS are referred to surgery, always with paranasal sinuses CT scan, as are those who have failed the clinical treatment for the disease. Patients with nasal polyposis above level II are also always referred to surgery.

It is hard to measure health and life quality issues. According to the World Health Organization, it encompasses physical, social and mental well being. On the other hand, social well being may change according to a country culture and development level. As we attempt to assess how much the disease impairs a patient’s life quality, we adapted the questionnaire for our population. By doing so, we have facilitated the very dialogue between interviewer and interviewee, besides obtaining a social assessment compatible with the environment where they live.

Two interesting studies were carried out using questionnaires to assess the life quality of patients with CRS. Damm’s^7^ study found post operative improvement in 85% of the cases, and Bhattacharyya’s^6^ study stressed the improvement of most symptoms in all patients, with greater emphasis in facial pain, congestion, nasal obstruction, rhinorrhea and headache.

In our study, we found general health improvements in 50% of the patients; and 54.2% of them said they enjoyed considerable gain in life quality. Notwithstanding, all the patients interviewed would recommend this procedure to a friend or relative, while only 3 would not undergo the surgery again if the problem recurred. When compared to the literature, we considered the life quality gain to be low. This may be due to the fact that chronic nasosinusal inflammatory diseases are frequently associated to a number of factors such as immune deficiencies, muco-ciliary alterations, vasomotor hyperactivity and atopy, which would definitely influence clinical outcome and surgical result. Alergic rhinitis was a problem reported by many of the interviewees, and it was known by all that such problem is not surgically treatable; its control should be carried out after surgery. At this point it is worth highlighting those low income patients often times do not follow post-operative prescriptions for anti-histamine and topical steroids. We must also bear in mind the environmental conditions where they live, with very little environmental control.

AS reported by other authors^6^, ^7^, ^12^, nasal obstruction, rhinorrhea and headache are the CRS symptoms that most impact life quality. In our study, these were also frequent complaints, especially when there were polyps associated. Nasal obstruction was present in 100% of the patients, headaches in 87.5% and rhinorrhea in 91.6%. Another very common symptom in our series, and not often reported in other papers, was hyposmia, which reached 83.3% of the patients. In approximately 36% of the patients, polyps may come from the olfactory mucosa, causing hyposmia, in our study; only one patient did not have any smell alteration. All other patients who complained of olfactory reduction had polyps, not only in the nasal roof, but in the entire nasal cavity, and the origin for hyposmia was both air flow obstruction and olfactory mucosa alteration.

Patients with nasosinusal polyps presented a certain trend toward more exuberant symptoms than those with CRS alone. All nasal polyp patients reported headaches, nasal obstruction, rhinorrhea and dry throat, and over 80% of them complained of smell alterations and post nasal drip. Such results should be analyzed considering that the study was carried out in a tertiary public hospital, where the difficult access to health services may delay diagnosis and to where the most severe cases are referred.

In Dursun’s^13^ study, where they tried to determine FESS success in CRS, polyps had 73.6% of post operative improvement, while recurrent polyposis had a 23.8% success rate. In general, FESS subjective success in treating CRS was reached in 83% of the cases, with 54.4% of the cases presenting associated nasosinusal polyposis.

In our study, symptoms that had the most improvement after surgery were nasal obstruction, headache and hyposmia. Polyp patients had a trend of presenting with greater symptoms relief after surgery. Nasal obstruction was present in both groups of patients, with a 92% improvement in patients with polyps (only one did not get better), and 72.7% in those with chronic nasosinusitis alone. Of the 10 patients with polyps who complained of headaches, seven improved, while in the 11 patients belonging to the CRS group only 6 improved. In CRS, there was a 20% improvement in dry throat, with only one patient reporting improvement among the five of them who had the symptom. In patients with nasal polyps, there was a 69.2% improvement rate, because 13 had dryness and nine improved. It is interesting to notice that besides the symptoms questioned, some patients with polyps revealed the stigma of a “growing meat leaving their nose”, which prevented them from leaving their homes because of prejudice. The improvement in these patients’ low self esteem make them report a greater surgical success than those with CRS alone, and it seems to us that the life quality improvement in cases of nasosinusal polyps goes beyond common symptoms improvement.

We highlight the fact that our study was carried out in a Public Hospital, where patients are somewhat used to long standing suffering of diverse origins and are not very demanding, and this makes it difficult to compare our data with those reported in the literature, which usually come from developed countries in different settings^14^. Other studies carried out in our country are necessary in order to assess the impact of surgical interventions on the life quality of our patients.

Our analysis tool did not offer us an objective measure of FESS efficacy, notwithstanding, our goal was to clarify whether or not there were clinical improvements in certain symptoms from each patient. We have sought subjective improvement, aware that this may not correspond to a “radiological” cure, but rather the patient’s satisfaction toward the surgical approach.

## CONCLUSIONS

Symptoms improvement attained with the endoscopic procedure has been satisfactory; however, the improvement magnitude was lower than what had been expected. The best results were achieved with nasal permeability and halitosis and cacosmia improvement after surgery. The low surgical improvement of some symptoms, such as rhinorrhea, may have been influenced, in our settings, by the presence of allergic rhinitis in patients with poor environmental control and the lack of nasal spray use. It is interesting to notice that those with nasal polyps reported more complaints in the pre-op and reported greater improvement in life quality, for most of the symptoms, when compared to CRS patients.
